# Water Soluble Fraction of Diesel Fuel Induced Histopathological Alterations in the Liver of *Channa punctatus*

**DOI:** 10.4103/0971-6580.75846

**Published:** 2011

**Authors:** Preeti Handa Kakkar, R. M. Saxena, N. S. Rathee, Mamta Joshi

**Affiliations:** Department of Zoology, D.A.V. (P.G.) College, Dehradun - 248 001, Uttarakhand, India

**Keywords:** Diesel fuel, fish, histopathology, liver, WSF

## Abstract

The aim of this work was to verify the effects of water soluble fraction (WSF) of diesel fuel in liver of *Channa punctatus*. The fishes were exposed to sublethal concentration of WSF of diesel (5%-DF1, 10%-DF2, 15%-DF3, 20%-DF4 and 25%-DF5) for 21 days. Significant histopathological lesions observed were dilation, congestion, thrombosis formation in hepatoportal blood vessel, melanomacrophage centers, hemolysis, hemorrhage, lymphocytic infiltration between the hepatocytes and necrosis & fibrosis in hepatocytes were the prominent changes in liver. The histological analysis showed increasing damages dose-dependents and time-dependents.

## INTRODUCTION

Pollution from industrial and petroleum- related activities are common in many parts of the world. Industrialization and urbanization are the main causes for such pollution. Furthermore, ever increasing number of vehicles and stationary engines has lead to fast growth of automobile workshops in city areas. The washing and servicing of engines and vehicles generate a large volume of oil-based wastes. Diesel used as fuel for automobiles and higher levels of aromatic hydrocarbons than that of crude oil are found in diesel and so the toxicity is higher for the diesel oil.[1] Aromatic hydrocarbons are more water soluble and disappear more slowly from solution compared to alkanes. They are accumulated by organisms in greater extent and retained longer than alkanes. These are the main factors contributing to high toxicity of diesel oil. The WSF of Diesel fuel present in water is known to affect the health of fishes and ultimately of human beings. Thus, it becomes necessary to assess the effect of WSF of diesel on fishes, because fish flesh is rich in protein and minerals like calcium, phosphorus and iron. Fishes are a very useful barometer of the real state of purity of water. No aquatic body should be considered in a satisfactory condition unless fish will live and thrive in it.

## MATERIAL AND METHODS

WSF was prepared as per the method given by.[2] Different concentration of Diesel fuel was denoted as DF1-5%, DF2-10%, DF3-15%, DF4 -20% and DF5-25%. *Channapunctatus* were collected from local rivers of Doon Valley and brought to the laboratory. After acclimatization the fishes were divided into 6 groups of about same size (15-18cm) and weight (60-80gm). A group of ten fishes was put in six different troughs, 1 serving as control and other 5 as experimental to study the effect of WSF on fishes. The fishes were exposed to various concentrations of WSF of Diesel for 21 days. The fish alive after 21 days was carefully removed from the test solution and sacrified immediately. For histopathological examination the liver were fixed in Bouins fluid for 24 hours followed by dehydration, embedding, sectioning and staining adopting the standard methods.[3] Detailed histopathological studies of control and treatment sections (5 microns) were made under microscope and photographs were taken.

## RESULTS AND DISCUSSION

**Figure 1:**
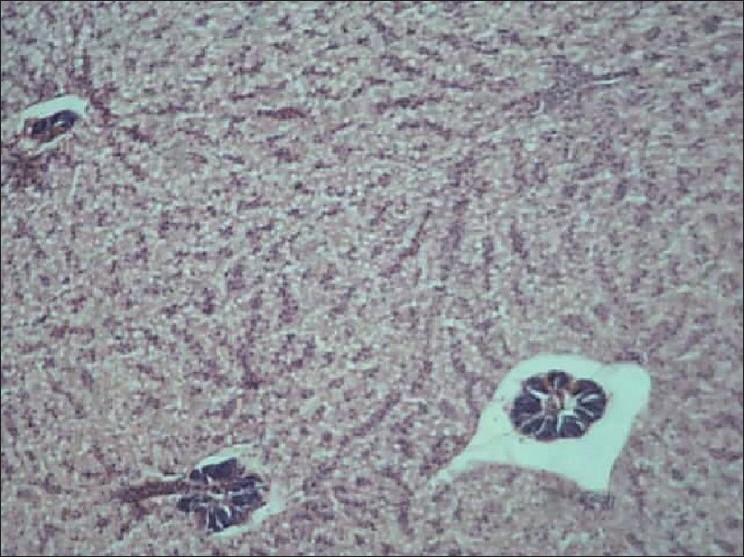
Liver showing hepatocytes with uniform nuclei and sinusuids (control group)

**Figure 2:**
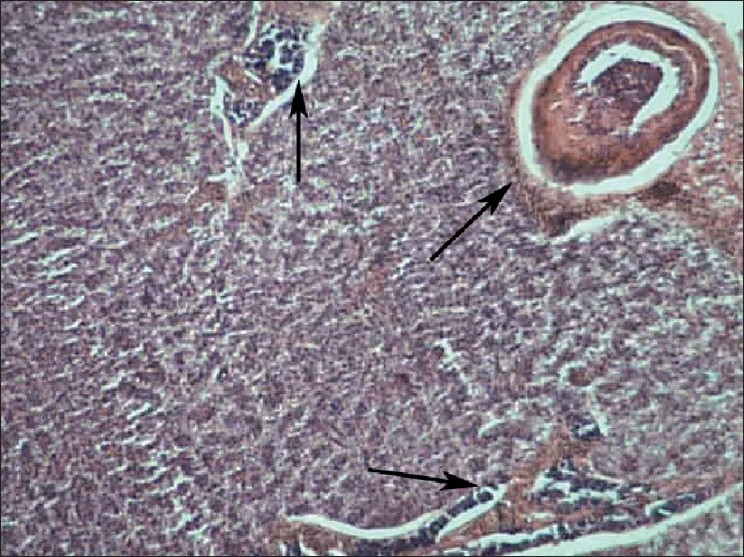
Liver showing degeneration of endothelial lining cells (2%- PF1) 100×

**Figure 3:**
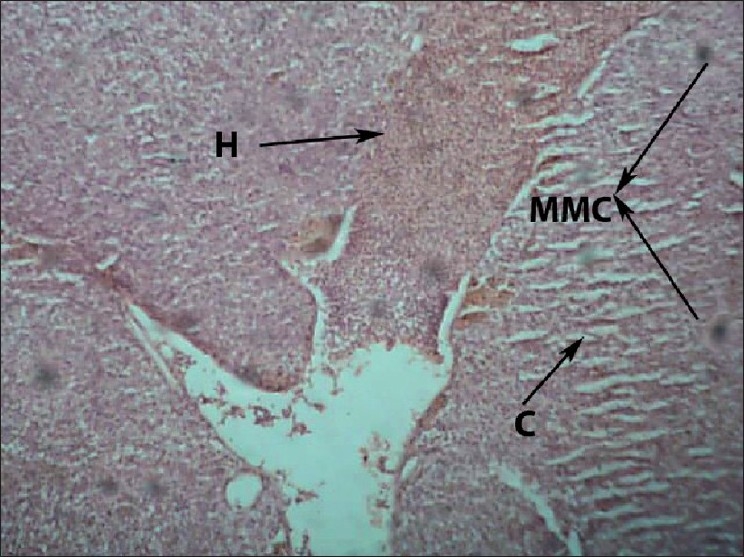
Liver showing marked swelling of hepatocytes and diffuse necrosis (4%-PF2) 100×

**Figure 4:**
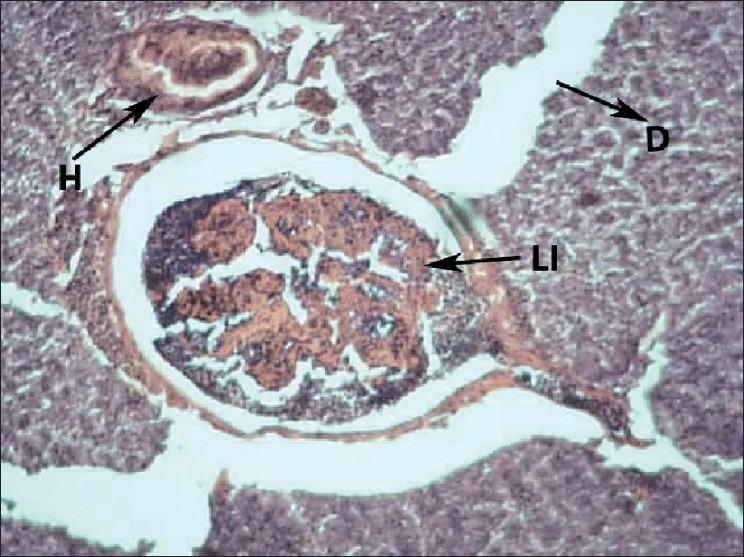
Liver showing hemolysis within the blood vessels (6%-PF3) 100×

**Figure 5:**
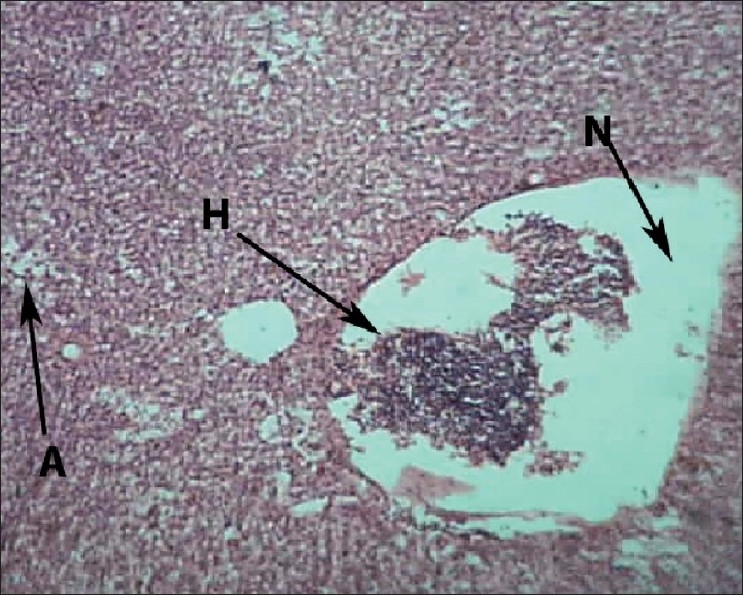
Liver showing dilation and congestion in blood sinusoids (8%-PF4) 100×

**Figure 6:**
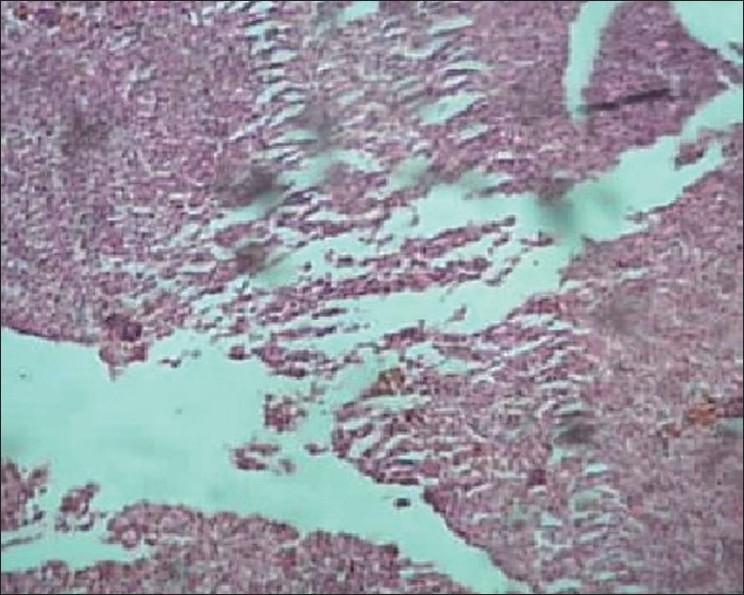
Liver showing coagulative necrosis (10%-PF5) 100×

Liver of control fish showed normal hepatic acini arrangement in regular manner. Hepatocytes have polygonal shape with clear cellular border lines and homogenous cytoplasm. The quite concentric nucleus has clear nucleoli [Figure 1]. Comparing the sections of the control and experimental fishes, dilation and thrombosis formation in hepatoportal blood vessel at 2%-DF1 [Figure 2]. The histopathological changes of the liver were more pronounced at 4%-DF2.There was melanomacrophage centers & congestion in blood sinusoids and intervascular haemolysis in hepatic blood vessels and hepatoportal blood vessels [Figure 3]. The normal architecture of liver tissue was markedly disrupted. Sinusoids in most cases were distended, dilation, hemorrhage and lymphocytic infiltration was observed at 6%DF3 [Figure 4]. With increasing the concentration hepatopancreas damage became more conspicuous and aggregation of inflammatory cells between the hepatocytes and focal area of necrosis was observed at 8%-DF4 [Figure 5]. The condition become more critical at higher concentration and Congestion and fibrosis between the hepatocytes were observed at 10%-DF5 [Figure 6].

The organ most associated with the detoxification and biotransformation process is the liver, and due to its function, position and blood supply[4] it is also one of the organ most affected by WSF contamination in the water.[5] Melanomacrophage Centres (MMC) recorded in the liver of exposed fish may be suggestive of metabolic disorders and it is commonly associated with dietary deficiency in response to WSF. Similar result were observed by[6] who observed the MMC in the liver of Molly fish after exposure to sodium perchlorate. The function of the MMC in the liver of fishes remains uncertain, but some studies have suggested that it is related to destruction, detoxification or recycling of endogenous and exogenous compounds.[7] Necrosis of some portions of the liver tissue that were observed probably resulted from the excessive work required by the fish to get rid of the WSF from its body during the process of detoxification by the liver. The inability of fishes to regenerate new liver cells may also have led to necrosis. The present results are in agreement with[8] who reported similar changes in the liver of *Astyanax sp*. exposed to WSF of crude oil. Further[9] reported, petroleum carcinogenic compounds caused necrosis in hepatopancreatic cell of *Palaemon serratus*. Haemolysis & Hemorrhage is a result of blood channel disruption and is indication of severe physical damage. The haemolysis, dilation, congestion, & fibrosis may be attributed to direct toxic effects of pollutants on hepatocytes, since the liver is the site of detoxification of all types of toxins and chemicals. These four alterations were not found earlier regarding with exposure of WSF of diesel fuel. The present result is in agreement with[4] who observed these alterations in *Tilapia zillii* and *Solea vulgaris* under the influence of different pollutants from Lake Qarun, Egypt. According to Saxena[10] lymphocytic infiltration in liver with round lymphocytes and dark basophilic nuclei were observed in fish after exposure to polluted water with heavy metals. Since the liver is usually the site of toxicant accumulation and detoxification, it is likely to show very extensive histopathology.

## CONCLUSION

In conclusion, the present study proved that WSF of diesel affected the histopathological changes of the liver of *Channa punctatus* and this effect was time dependent. This may not result in fish kill immediately but definitely represents a health hazard to human consumers. Therefore it is important that the waste water should be treated before pouring in to the water bodies. Hene, Government should take remedial measures and pass appropriate legistation.
